# Discovery, Optimization, and Clinical Application of Natural Antimicrobial Peptides

**DOI:** 10.3390/biomedicines9101381

**Published:** 2021-10-03

**Authors:** Armando A. Rodríguez, Anselmo Otero-González, Maretchia Ghattas, Ludger Ständker

**Affiliations:** 1 Core Facility for Functional Peptidomics, Ulm University Medical Center, 89081 Ulm, Germany; 2 Core Unit of Mass Spectrometry and Proteomics, Ulm University Medical Center, 89081 Ulm, Germany; 3 Faculty of Biology, University of Havana, Havana 10400, Cuba; aotero@fbio.uh.cu; 4 Faculty of Pharmacy and Biotechnology, German University in Cairo (GUC), Cairo 11511, Egypt; mghattasfakhery@gmail.com

**Keywords:** antimicrobial, peptide, conjugate, nanoparticle, delivery system

## Abstract

Antimicrobial peptides (AMPs) are widespread in multicellular organisms. These structurally diverse molecules are produced as the first line of defense against pathogens such as bacteria, viruses, fungi, and parasites. Also known as host defense peptides in higher eukaryotic organisms, AMPs display immunomodulatory and anticancer activities. During the last 30 years, technological advances have boosted the research on antimicrobial peptides, which have also attracted great interest as an alternative to tackling the antimicrobial resistance scenario mainly provoked by some bacterial and fungal pathogens. However, the introduction of natural AMPs in clinical trials faces challenges such as proteolytic digestion, short half-lives, and cytotoxicity upon systemic and oral application. Therefore, some strategies have been implemented to improve the properties of AMPs aiming to be used as effective therapeutic agents. In the present review, we summarize the discovery path of AMPs, focusing on preclinical development, recent advances in chemical optimization and peptide delivery systems, and their introduction into the market.

## 1. Introduction

Sir Alexander Fleming is world renowned for the discovery of the antibiotic penicillin in 1928. However, a few years earlier (1922), he had discovered an antimicrobial protein when he found a bacteriolytic substance from nasal secretion, which he called “Lysozyme”. In addition, he described it as “very widespread in nature”, after finding it to be present in other body fluids and tissues, as well as in other animals and plants [1]. In fact, to date, antimicrobial peptides and proteins (AMPs) have been isolated from a variety of sources such as humans (and other mammals), amphibians, fishes, reptiles, birds, crustaceans, myriapods, spiders, scorpions, mollusks, protozoa, and plants [2]. AMPs can display antibacterial, antiviral, antifungal, and antiparasitic activities. In addition, AMPs are considered to be universal defense molecules of the innate immune system either in vertebrate or invertebrates; therefore, they are also grouped within the terms “host defense peptides” and “innate immune peptides”, which cover their antimicrobial and immune modulatory functions as well as other functional roles [3].

In general, natural AMPs are ribosomally synthesized polypeptides composed of 12–50 amino acid residues [4]; they can also be proteins (mainly exhibiting antibacterial activity) of 100–300 amino acid residues [5], as well as acidic peptides [6]. APD3 https://wangapd3.com/main.php (accessed on 21 April 2021) [7], a comprehensive database of natural antimicrobial peptides, contains 3257 annotated peptides (mostly antibacterials). Most of them are composed of 20–50 amino acid residues, with those peptides of about 30 amino acid residues being the most abundant group. Regarding their net charges, basic peptides are widely dominant, with charges of more than +10 at physiological pH, with those of charge from +2 to +4 being the most abundant ones. This is due to the higher abundance of basic residues than acidic residues; on average, Lys and Arg are threefold more abundant than Glu and Asp residues. The presence of hydrophobic residues is also common, which in many cases represent about 50% of the total number [8], with Leu being the most frequent one. In addition, disulfide bridges and alpha-helices are among the most common structural features found in AMPs [9]. In general, they can be classified as α-helical peptides, β-sheet peptides, and extended/flexible peptides; this last group includes Pro-rich, Arg-rich, Lys-rich, and His-rich peptides, among others [10].

The rapid growth of the information on new antimicrobial peptides has prompted scientists to classify and organize AMPs in specialized databases such as APD3, https://wangapd3.com/main.php (accessed on 21 April 2021) [7], DRAMP http://dramp.cpu-bioinfor.org/ (accessed on 21 April 2021) [11], DBAASP https://dbaasp.org/ (accessed on 21 April 2021) [12], SATPdb http://crdd.osdd.net/raghava/satpdb/ (accessed on 21 April 2021) [13], and CAMP_R3_ http://www.camp3.bicnirrh.res.in/ (accessed on 21 April 2021) [14], which contain thousands of AMPs sequences of natural origin (ribosomal/nonribosomal) exhibiting not only antimicrobial activity but also anticancer, antidiabetic, wound healing, chemotactic, anti-inflammatory, spermicidal, insecticidal, ion channel inhibition, protease inhibition, and antioxidant activities [7]. In addition, some of these databases include synthetically constructed peptides with validated antimicrobial activity and others whose activity has only been predicted [14]. Other databases are more specific, such as AVPdb http://crdd.osdd.net/servers/avpdb/ (accessed on 21 April 2021) [15], focused on antiviral peptides; YADAMP http://www.yadamp.unisa.it (accessed on 21 April 2021) [16] for antibacterial peptides; PlantAFP http://bioinformatics.cimap.res.in/sharma/PlantAFP/ (accessed on 21 April 2021) [17], focused on plant antifungal peptides; BACTIBASE http://bactibase.hammamilab.org/main.php (accessed on 21 April 2021), a data repository of bacteriocin natural antimicrobial peptides [18]; and LantiBase https://lantibase.weebly.com/ (accessed on 21 April 2021), a lantibiotic structure database. In addition, ADAPTABLE http://gec.u-picardie.fr/adaptable (accessed on 21 April 2021) [19] and LAMP2 http://biotechlab.fudan.edu.cn/database/lamp/ (accessed on 21 April 2021) [20] comprise merged data or links to other AMP databases, allowing comprehensive searches. A recent review summarizes the publicly available databases of AMPs and related prediction tools [21].

Antibacterial peptides can kill bacterial pathogens by membrane permeabilization, also by acting on the bacterial cell wall and intracellular targets. Besides directly killing bacteria, these peptides can recruit and activate immune cells [10]. Antiviral peptides can block viral infection by acting on viral transcription and replication enzymes, such as proteases and polymerase, or by inactivating viral structural proteins. Antiviral peptides can also target host cell factors involved in the replication cycle or can act as immunomodulators such as interferons and gamma globulins [22,23]. Antifungal peptides can inhibit the biosynthesis of cell wall components such as Chitin or inhibit the biosynthesis of 1,3-β-Glucans, which are essential for cell wall integrity. Moreover, they can exert selective activity on membranes by inducing membrane permeability and pore formation and targeting intracellular components such as the mitochondrial membrane, thus provoking a signaling cascade that leads to cell death [24]. Most of the antiparasitic (antiprotozoal) peptides act by altering the membrane integrity and inducing osmotic cell lysis. By contrast, others modify the fluidity of protozoal membranes, impairing the activity of membrane-bound proteins. Another mechanism involves translocating the antiprotozoal peptide into the cell and interacting with intracellular targets, causing metabolic and bioenergetic pathways to collapse. In addition, some peptides induce protozoal death by triggering autophagic- or apoptotic-like processes [25]. Although the term antimicrobial refers to antibacterial, antiviral, antifungal, and antiparasitic activities of AMPs, not all AMPs possess broad-spectrum activity. Of the total number (3257) of AMPs in the APD3 database https://wangapd3.com/main.php (accessed on 21 April 2021) [7], those having at least antibacterial activity are dominant by a wide margin, reaching the number of the 2727 (84% of the total number); 1032 peptides (31%) possess both antibacterial and antifungal activities; 74 peptides (2.2%) have antibacterial, antifungal, and antiviral activities; and only 13 AMPs (0.4%) have antibacterial, antifungal, antiviral, and antiparasitic activities.

**Figure 1:**
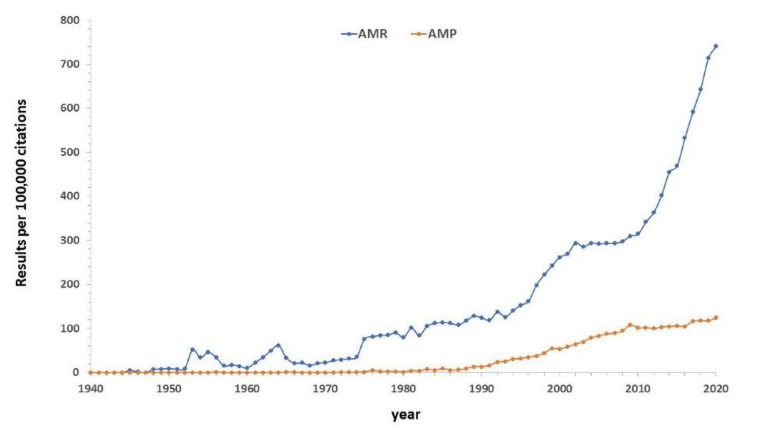
Publication trend related to antimicrobial resistance (AMR, blue trace) and antimicrobial peptide (AMP, red trace) in Pubmed during the period 1940–2020. Search terms used for AMR: drug resistance, antimicrobial resistance, or antibiotic resistance either in the title or abstract. Search terms used for AMP: peptide and (antimicrobial or antibacterial or antiviral or antifungal or antiparasitic) either in the title or abstract. For a suitable comparison, every set of data was normalized to the number per 100,000 results/year from Pubmed. The search was conducted by Pubmed by Year (https://esperr.github.io/pubmed-by-year/ (accessed on 5 September 2021)). Both traces represent an increasing interest in AMR and AMP, especially during the last 30 years. Concern about AMR has boosted the research on the topic to have an exponential growth.

The period between 1940 and 1960 was markedly successful regarding the discovery and application of many antibiotic compounds. This led to a loss of interest in the therapeutic potential of host defense peptides/proteins (such as lysozyme) provided by the immune adaptative system, which were known to protect the humans and other living creatures, against microbial infections [6]. Although antimicrobial resistance (AMR), commonly referred to as drug resistance or antibiotic resistance, was already known in 1940 [26], a very flourishing pharmaceutical industry seemed to be up to the challenge during the so-called “golden era of antibiotics discovery”. However, in the 1960s, the former optimism turned into an increasing concern about antimicrobial resistance [27], leading to a growing interest during the last 30 years (Figure 1). AMR happens when bacteria, viruses, fungi, and parasites no longer respond to medicines, making infections harder or even impossible to treat. Therefore, AMR increases the risk of disease spread, severe illness, and death [28]. Significant relevance has acquired drug resistance from bacteria *Escherichia coli, Klebsiella pneumoniae, Staphylococcus aureus,* *Mycobacterium tuberculosis*, human immunodeficiency virus (HIV), the malaria parasite *Plasmodium falciparum,* and the fungus Candida auris. The World Health Organization considers antimicrobial resistance among the top 10 global public health threats facing humanity [28]; consequently, the search for novel antimicrobial agents represents an urgent matter.

Antimicrobial peptides (AMPs) are considered as an alternative to overcoming drug resistance encountered by the use of conventional antibiotics, since antimicrobial resistance and host cationic antimicrobial peptides have coevolved in nature. The combination of several antimicrobial mechanisms in one molecule, the action on essential nonprotein bacterial structures, and the presence of cationic AMPs in high concentrations at sites of infection could have prevented bacteria from developing highly effective AMP resistance. In addition, structural evolution through the natural design of stabilizing disulfide bridges, extensive mutations of AMP sequences, and adaptation of their electrostatic properties might contribute to eluding antimicrobial resistance [29]. Increasing interest in antimicrobial peptides is reflected in the number of publications in the last 30 years (Figure 1).

The path has been long from those starting works that provided the first clues on the existence of antimicrobial peptides to nowadays when in much shorter times a huge number of peptides are discovered and synthetized, modified, or completely designed for optimal activity, and their delivery systems improved for the most efficient action. In this sense, the present review article aims to show the evolution of the discovery of antimicrobial peptides and the latest advances in some strategies aimed to improve AMP suitability for their use in humans. Such strategies include the introduction of chemical modifications and delivery systems such as liposomes and nanoparticles. In addition, some aspects to consider in preclinical studies are discussed and examples of AMPs introduced into the market.

## 2. Discovery of Native AMPs: A Historical Overview

Although research on AMPs became relevant in the 20th century, during the last 15 years of the 19th century, some reports demonstrated the existence of antimicrobial substances in blood, leucocytes, and lymphatic tissues, with activity against Gram-positive and Gram-negative bacteria [30]. Unfortunately, technologies back in those days were insufficient to accurately describe such antimicrobial peptide agents, including their chemical structures. Logically, such observations were supported by later rediscoveries and more comprehensive biological and chemical studies of these substances.

During the first decades of the past century, low-resolution techniques such as extraction, precipitation, centrifugation, and crystallization were commonly used for the isolation and purification of bioactive molecules, including AMPs. Lysozyme was discovered in 1922, and early attempts to obtain it in its pure form from hen egg-white lysozyme gained importance in 1927–1937, when several preparations based on precipitation procedures were reported. In 1937, highly pure lysozyme was obtained and crystallized [31,32], which allowed further studies of this antimicrobial protein. However, only after many years, in the 1960s, the primary structure of egg-white lysozyme was finally elucidated [33].

In 1928, the first study on a bacteriocin (Nisin A) was published [34]. This peptide was discovered in *Lactococcus lactis* for its inhibitory effect on *Lactobacillus bulgaricus* growth. However, only after many years, Nisin A was found to be composed of several similar peptides, successfully separated by countercurrent distribution and their amino acid composition analyzed by paper chromatography [35]. Nisin A is the first known member of the Nisin family of AMP; it has been widely used as a food preservative given its broad-spectrum bacteriocin against mostly Gram-positive foodborne bacteria. The nisin family of lantibiotic peptides contains several unusual amino acid residues and five lanthionine rings due to enzymatic post-translational modifications [36].

Between 1939 and 1941, a mixture of peptides named tyrothricin (gramicidin and tyrocidine) was isolated by precipitation in diluted HCl, extraction with organic solvents, and recrystallization, starting from an autolyzed cell culture of *Bacillus brevis* [37]. Although gramicidin was found to be active only against Gram-positive bacteria, its activity was much higher than tyrocidine, which was also active against Gram-negative bacteria [38]. Therefore the action of tyrothricin (gramicidin + tyrocidine) against Gram-positive bacteria mainly was attributed to gramicidin in the topical treatment of wounds infected with Gram-positive bacteria [39]. Later, gramicidin was described as a mixture of peptides and gramicidins A–C [40], which were sequenced in the 1960s [41,42,43]. Gramicidins are considered as the first antimicrobial peptides clinically tested [44], commercially manufactured, and with a known amino acid sequence [45].

During the 1940s and the 1960s, significant advances in the separation and analysis of peptides started to accelerate the discovery and characterization of novel bioactive molecules, including antimicrobial peptides. In 1941, Martin and Synge developed a new theory of chromatography and introduced partition chromatography, which represented a breakthrough in separation sciences [46]. In addition, paper chromatography was introduced [47], years later being replaced by thin-layer chromatography. In 1945, Sanger introduced a method for N-terminal peptide sequencing, which was superseded by other C-terminal and N-terminal sequencing methods, especially Edman degradation, developed by Edman and coworkers in 1950 [48] who years later implemented an automated version of this technique [49]. Between the end of the 1940s and early 1950s, Moore and coworkers developed the amino acid analysis technique [50]. In 1963, Merrifield and colleagues developed solid-phase peptide synthesis [51]. These technological advances and subsequent modernization in later years shortened the time gap between the initial studies from antimicrobial’s sources and the separation, sequencing, synthesis, and structure analyses of antimicrobial peptides.

In 1942, purothionin, the first member of the antimicrobial peptide family of thionin, was isolated from wheat endosperm *Triticum aestivum* and found to inhibit the growth of a *Pseudomonas solanacearum* and *Xanthomonas campestris* [52]. It was not until the 1970s that the primary structure of two purothionins was elucidated [53,54]. Thionins are cysteine-rich basic peptides of molecular masses about 5 kDa, which exhibit toxicity against bacteria, fungi, and yeast. Thionins are classified into five groups; purothionins belong to group I. [55]. Nowadays, it is known that thionins and other AMPs distributed across the plant kingdom are an integral part of the immune system in plants [56].

In the years 1942–1944, soviet gramicidin (gramicidin S), the first known circular AMP peptide from bacteria (*Bacillus brevis*, a different strain than the one where Gramicidin A–C were isolated), was discovered and clinically applied in the treatment of infected wounds [57,58,59].

In 1946–1947, nonribosomal AMPs polymyxins B and E (also known as colistin) were discovered from the soil bacterium *Paenibacillus polymyxa* [60,61]. Colistin and polymyxin B belong to the primary classes of antibiotics with activity against the most common Gram-negative bacteria [62]. Polymyxins are basic cyclic peptides. Polymyxin E is composed of colistin A and B, whereas polymyxin B is composed of polymyxin B1 and B2; their molecular masses are about 1.2 kDa. During the 1950s and 1960s, polymyxins were used for the treatment of infections caused by Gram-negative bacteria. However, in the 1970s, their use was reconsidered because of concern for nephrotoxicity; therefore, they were replaced by novel, more active, and less toxic antibiotics [62]. The emergence of Gram-negative bacteria resistant to most antimicrobial agents has led to a resurgent interest in polymyxins. WHO included colistin with the highest priority in the list of “critically important antimicrobials for human medicine”, for the treatment of infections from multidrug-resistant Gram-negative superbugs, such as those of the family *Enterobacteriaceae* [63].

During the 1960s, antimicrobial peptides from animals gained attention with the discovery of melittin, a peptide toxin representing the main component of bee venom and its principal pain-producing substance [64]. Melittin is an antimicrobial peptide with antibacterial and antifungal activities [65,66]. In addition, during the 1960s, another toxic peptide was isolated from the skin secretion of *Bombina* species [67]. Later, this hexapeptide was shown to be the C-terminus of bombinin, fully sequenced in 1970 [68]. Since then, several other antimicrobial peptides of the bombinin family have been discovered from Bombina skin secretion, with activity against many Gram-negative and Gram-positive bacteria, as well as fungi. Frogs commonly inhabit a humid environment; the function of these antimicrobial peptides is to prevent microbial growth on the skin [69].

In the 1970s, the evolution of separation, analysis, and sequencing techniques impacted the discovery of antimicrobial peptides. The introduction of high-performance liquid chromatography (HPLC) considerably improved separations with higher resolution, reproducibility, and sensitivity in short run times. Among the chromatographic techniques, reversed-phase HPLC has become one of the most widely used for peptide separation [70]. Protein/peptide and DNA purification and analysis on gel were also improved with the introduction of modern SDS-PAGE [71] and separation on agarose gel with ethidium bromide stain [72]. Novel and faster DNA sequencing methods were developed [73,74], and recombinant DNA technology was introduced [75].

In 1970, the first member of cyclotides (cyclic peptides), kalata B1, was discovered from the plant *Oldenlandia affinis* [76]. However, after more than 20 years, its primary structure and three-dimensional structure were elucidated [77]. Cyclotides are peptides with resistance to high temperatures, such as in boiling water; their peculiar three-dimensional circular structure confers such stability. Kalata B1 initially drew attention for its uterotonic activity as an ingredient of kalata-kalata, an African medicinal extract of *Oldenlandia affinis* used to assist in childbirth [78].

Late in the 1970s and during the 1980s, the continuous development of computer science in parallel with advances in biology set the path to overcoming future challenges such as analyzing large datasets, such as complete genomes and proteomes [79]. In the 1980s, a breakthrough in biomolecule analysis was achieved by both the application of NMR (nuclear magnetic resonance) [80] to peptide structure elucidation and the development of mass spectrometry ionization methods ESI (electrospray source ionization) and MALDI (matrix-assisted laser desorption/ionization), which made possible the molecular mass analysis of large biomolecules such as proteins and greatly improved peptide/protein sequencing [81,82] in later years.

In 1980–1981, a new class of antimicrobial peptides was found, cecropins, from pupae of the cecropia moth *Hyalophora cecropia* [83]. Cecropins P9A and P9B were isolated by a combination of ion-exchange steps, and their primary structures were elucidated by amino acid analysis, Edman degradation, and carboxypeptidase Y degradation [84]. Cecropins are important components of the innate immune system of insects [85]; they are induced by bacterial infection and exhibit potent bacteriolytic activity against *E. coli* and other Gram-negative bacteria; they are also active against Gram-positive bacteria [83]. Cecropins were initially discovered in insects, but later, they were shown to be widespread in the animal kingdom [85]. This landmark work on cecropins attracted great interest in innate immunity research [3].

In 1985, the antimicrobial peptides HNP1, HNP2, and HNP3 were discovered in normal human neutrophils. These peptides are considered part of the innate immune system and were the first alpha-defensin found in mammals. HNP1-3 contain 29–30 amino acid residues, including Cys residues that form three disulfide bridges [86]. The mixture of these peptides killed *Staphylococcus aureus, Pseudomonas aeruginosa,* and *Escherichia coli* when tested in vitro. The combination also exhibited antifungal properties and inactivated herpes simplex virus, Type 1 [87].

In 1987, magainin 1 and 2 were chromatographically (IEX and RP-HPLC) purified and sequenced from the skin of the African clawed frog *Xenopus laevis*. The use of cDNA techniques allowed the discovery of a precursor fragment of these peptides with no homology in databanks. Magainins are closely related 23 amino acid residues peptides that represent a previously unrecognized class of vertebrate antimicrobial peptides exhibiting a broad-spectrum antimicrobial activity against numerous bacteria, fungi, and protozoa [88].

In the late 1980s and early 1990s, further contribution to the knowledge on AMPs in mammalian innate immune defense against invasive bacterial infection was brought by the discovery of the first cathelicidin in mammalian myeloid cells [89]. A dodecapeptide named bactenecin exhibiting bactericidal activity against *Escherichia coli* and *Staphylococcus aureus* was purified and sequenced from an acid extract of bovine neutrophil granules [90]. This peptide has a cyclic structure due to the formation of a disulfide bond. A three-dimensional structure was proposed by computer modeling. In addition, in those years, salivary peptide histatins 1, 3, and 5 were discovered. These peptides showed fungistatic effects on *Candida albicans* and were chromatographically purified and characterized from human parotid secretion. The complete amino acid sequences of histatins were determined by automated Edman degradation, *Staphylococcus aureus* V8 protease, and tryptic peptides [91].

Humoral innate immunity in honeybees are mainly constituted by antimicrobial peptides which are constitutive or elicited in the presence of infectious agent [92]. In 1989, apidaecins (Ia, lb, and II), a new family of peptide antibiotics, was discovered by inducing their production in honeybee lymph upon bacterial infection. Apidaecins were purified, sequenced, and synthetized, and their structures were determined by NMR. They were described as cationic nonhelical peptides, highly stable at a low pH and high temperature, apparently due to their unique Pro residue content (33%). Apidaecins showed bacteriostatic rather than lytic activity against many plant-associated bacteria and some human pathogens [93]. These are short proline-rich peptides that contain 18 amino acid residues. Their C-terminus is linked to the antimicrobial action, which is lost when a different amino acid residue is introduced. Apidaecins present a variable N-terminus with a spectrum of antimicrobial activity after sequence modifications, which is suitable for protein engineering depending on the characteristics of a particular pathogen [94,95]. Other AMPs found in honeybee are abaecin [96], hymenoptaecin [97], and defensins [98,99].

From the 1990s to date, the number of antimicrobial peptides discovered from natural sources increased drastically compared to previous decades. For brevity, only some examples are mentioned in the present work; a timeline list containing the most relevant examples can be found in the APD3 database [7].

In 1991, PR-39, a new proline-rich peptide (49% Pro residues) was isolated from a thermostable extract of pig small intestine by precipitation and subsequent chromatographic steps [100]. The pure peptide was analyzed by mass spectrometry, and its sequence was elucidated by digestion with trypsin, carboxypeptidase Y, fragments separation by RP-HPLC, and capillary-zone electrophoresis, followed by Edman degradation. PR-39 exhibited antibacterial activity against several Gram-negative and Gram-positive bacteria [100]. Its elevated arginine content and the consequent positive charge contribute to the antimicrobial activity [101]. In addition, its high proline content confers resistance to degradation by serine proteases, and its polyproline structure contributes to the inhibition of bacterial DNA and protein synthesis [102]. Further research indicated that PR-39 is involved in several other functions such as promoting angiogenesis, wound healing, and leukocyte chemotaxis [103].

In addition, in 1991, tracheal antimicrobial peptide (TAP), a cysteine-rich 38-residues peptide with antibacterial and antifungal activity, was isolated from mammalian tracheal mucosa. This peptide, considered the first known beta-defensin, was chromatographically purified and sequenced by a combination of Edman degradation and cDNA analysis. Authors found that the mRNA encoding this peptide is more abundant in the respiratory mucosa than in whole lung tissue, providing evidence of the role of this tissue in host defense [104]. TAP was found to be a potent bactericidal against both Gram-positive and Gram-negative bacteria. In addition, a domain analysis using synthetic peptides showed that the minimum functional domain of the bactericidal activity in TAP is composed of the last 17 aa residues [105].

In 1995, the first human cathelicin-like peptide (LL-37, formerly known as FALL-39) was discovered by screening a human bone marrow cDNA library using a PCR probe derived from the PR-39 gene. RNA blot analyses revealed that LL-37 is expressed mainly in human bone marrow and testis [106], also in skin, especially in case of injury provoked by trauma, infection, or inflammation [107]. The synthetic peptide was initially found to be active against *Escherichia coli* and *Bacillus megaterium* [106]; further research described its immunomodulatory role by inducing cytokine production and attracting and regulating the activity of immune cells [108].

In 1999, RTD-1 (rhesus theta defensin 1), a novel cyclic antimicrobial peptide, was isolated from primate leukocytes. RTD-1 was isolated and sequenced by proteolytic digestion and further Edman degradation and MALDI-TOF MS. This peptide showed microbicidal activity against bacteria and fungi at low micromolar concentrations, and its cyclic (head to tail) form was three times more active than the acyclic one. RTD-1 is formed through ligation of two truncated α-defensins, which demonstrates the existence of a posttranslational processing pathway that produces head-to-tail peptide chain ligation primate cells [109]. Further research showed the presence of θ-defensin RTD-2 to 6 in neutrophil of rhesus monkeys, being less abundant than RTD-1. Neutralization of θ-defensins with antibodies as well as the supplementation of human granule extracts with RTD-1 demonstrated a prominent microbicidal role for θ-defensins [110].

In 2001, a new antimicrobial peptide (DCD-1) with no homology to any known AMP was purified from sweat glands and sequenced by Edman microsequencing and nanoelectrospray-tandem mass spectrometry. DCD-1 was active against *E. coli, E. faecalis, S. aureus,* and *C. albicans*. RT-PCR analysis showed that the precursor (Dermcidin, DCD) is highly expressed in human skin, melanocytic nevus tissue, and cutaneous melanoma tissue. These findings indicated that sweat plays a role in the regulation of human skin flora through the presence of DCD-1, thus being part of the innate immune response of the skin [111]. Unlike most well-studied AMPs, DCD-1 is longer (49 aa residues) and has a negative net charge in physiological conditions [112]. Its antimicrobial mechanism comprises the formation of ion channels of unique features in the membrane of bacteria [113].

In 2005, Plectasin, the first defensin AMP from fungus (*Pseudoplectania nigrella*), was discovered. Plectasin was initially identified from a cDNA library and then recombinantly expressed in *Aspergillus oryzae* high-efficiency protein expression system. The peptide was purified by CEX, and its expected molecular mass was confirmed by LC–ESI–QTOF. Plectasin 3-D structure was determined by NMR. This peptide was active against a large number of Gram-positive bacteria [114]. Plectasin was also found to inhibit human voltage-gated potassium channels (K_v_) in a similar mode compared to some known animal toxins [115]. K_v_ channels are involved in many pathological processes [116]; therefore, this finding may open new opportunities for the use of Plectasin.

In 2012, a role for human keratins in epithelial innate immunity as a source of endogenous antimicrobial peptides was suggested after the discovery of K6A-derived peptides with bactericidal activity. These Glycine-rich short peptides (in general <3kDa) were obtained from human corneal epithelial cells and then fractionated by size and sequenced by LC-MS/MS. These AMPs are likely to have been released by proteolytic digestion of keratin K6A and belong to the region within the residues 515–555, located in the C-terminus [117]. Further research demonstrated that extracellular bacterial ligands enhance phosphorylation of several Ser residues in K6A provoking a keratin filament depolymerization. Soluble K6A is then ubiquitinated and targeted to the ubiquitin-proteasome system for degradation, thus generating the AMP fragments in human corneal epithelial cells [118,119].

Similarly to KA6, other naturally occurring human protein fragments have been identified as antimicrobial (mostly antiviral) peptides, such as a CC Chemokine 1 fragment, an alpha 1-antitrypsin fragment [120], a serum albumin fragment [121], and a cystatin C fragment [122], all of them isolated from human hemofiltrate, as well as hemoglobin fragments isolated from placenta [123,124].

In 2015, the use of ichip for high-throughput in situ cultivation of “uncultivable” microbial species (developed in 2010 [125]) allowed the identification of a novel antimicrobial peptide, Teixobactin. The peptide was isolated from the new species of β-proteobacteria *Eleftheria terrae* and then purified by reversed-phase chromatography and analyzed by mass spectrometry. Teixobactin is a novel depsipeptide that contains enduracididine, methylphenylalanine, and four d-amino acid residues. This depsipeptide showed excellent activity against Gram-positive bacteria; no mutants of *Staphylococcus aureus* or *Mycobacterium tuberculosis* resistant to teixobactin were obtained [126]. Unlike other antibiotics, Teixobactin is a cell wall inhibitor that binds lipid II and III instead of proteins; therefore, the development of resistance by mutations of the target should be minimal, which may pave the road toward developing novel antibiotics that avoid the development of resistance [127,128].

Another AMP with an unusual structure, which targets important Gram-negative bacteria, was discovered in 2019. Darobactin was isolated by bioassay-guided purification from *Photorhabdus khaini.* Its structure, comprising seven amino acid residues with two macrocycle crosslinks, was elucidated by mass spectrometry and NMR analysis [129]. Darobactin targets BamA chaperone at the outer membrane by backbone contacts, which are particularly robust against potential resistance mutations [130]. Darobactin represents a promising lead compound for developing therapeutic agents that act on surface targets of Gram-negative pathogens and do not require penetrating across their permeability barrier [129].

Despite the growing interest in AMPs, the use of these natural molecules in clinical trials can be challenging in most cases due to their proteolytic digestion by enzymes, short half-lives in vivo, and cytotoxin profile upon oral/systemic administration. Chemical modifications and the use of delivery systems have been implemented to improve the properties of AMPs and, therefore, to overcome such limitations [10]. Chemical modifications include the use of d-amino acids, cyclization, acetylation, and peptidomimetics to elude proteolytic digestion, as well as sequence shortening and replacement by other residues to increase activity and reduce production costs. On the other hand, inorganic and polymer materials, surfactant/lipid self-assembly systems, and peptide self-assembly systems are among the delivery systems (to which the AMP is covalently bound or noncovalently encapsulated) used to improve the stability, toxicity, half-life, and release profile of AMPs [10]. In the following sections, we discuss some of the strategies used to improve the properties of AMPs.

## 3. Derivatives and Optimization: SAR Analysis

Treatment of bacterial infections continues to be a challenge due to antimicrobial resistance. Natural antimicrobial peptides may offer a new option for treating of bacterial infections, but several factors limit their clinical utility.

**Figure 2:**
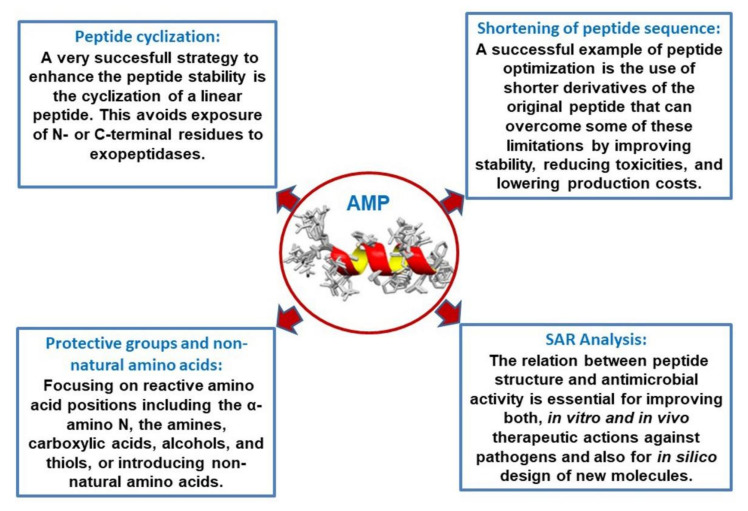
Different key strategies to optimize the structure–activity relationship of an AMP.

The activity of natural antimicrobial peptides (AMPs) primarily is based on their cationic net charge and their amphipathic fold, which allow their interaction with the negatively charged membranes of microorganisms leading to cell lysis, commonly through membrane disruption [131]. Other AMPs can translocate across the membrane and kill microorganisms by interacting with intracellular targets [132]. In this section, peptide optimization by cyclization, derivatization, sequence shortening, and SAR analysis are commented on (Figure 2).

Although many AMPs exhibit a strong antimicrobial effect in vitro, this effect can be severely affected under physiological concentrations of salts, serum proteins, divalent cations, and glycosaminoglycans, which are commonly found in mammalians body fluids [133]. A further critical point for clinical applications of AMPs is their susceptibility to proteases, which is directly related to their stability in biological fluids and tissues and their plasma half-life. For example, the direct clinical use of LL-37 is limited due to its susceptibility against endogenous enzymes found in the gut (trypsin, pepsin), pancreas (elastase), and serum (plasmin) [134,135]. In most cases, the direct application of a naturally found peptide sequence is limited by its missing oral availability, its low stability, high production costs of longer sequences, possible toxicity on eukaryotic cells, and, in many cases, low potency. Therefore, the optimization criteria of natural peptide sequences are in most cases: (a) enhancement of potency, (b) reduction of putative toxicity, (c) shortening of peptides length, and (d) improvement of peptide stability.

### 3.1. Shortening of Peptide Sequence

A successful example of peptide optimization was the use of shorter derivatives of LL-37. These shorter isoforms of LL-37 showed improved stability and reduced toxicity [136]. Moreover, the costs of production were significantly lower. This study by Shurko et al. [136] showed that the truncated derivatives of LL-37, LL-13, and LL-17 showed, alone and in combination with vancomycin, an enhanced activity against clinically relevant bacteria, e.g., drug-resistant *S. aureus* strains including methicillin-resistant *S. aureus* (MRSA) and vancomycin-resistant *S. aureus* (VRSA) strains in vitro. In combination with vancomycin, LL-13 and LL-17 demonstrated synergistic effects against VRSA and were able to recuperate the sensitivity against vancomycin after pretreatment. Moreover, LL-13 and LL-17 exhibited potent activity against the development of biofilms by *S. aureus*.

Other potent and promising LL-37 peptide fragments have been designed and were successfully applied in human clinical studies, e.g., OP-145, previously termed P60 4Ac. Nell et al. [137] scanned the LL-37 sequence with a window size of 22–25 mer identifying a 24 mer (amino acid sequence 13–36) as the most promising segment in terms of antimicrobial activity and with similar efficacy as LL-37 in terms of LPS and LTA neutralization and lower proinflammatory activity. This peptide was then optimized particularly at the C-terminal part of the peptide to favor the formation of an ideal amphipathic helix. For improvement of the stability of the peptide against proteolytic degradation, the N- and C-terminus of OP-145 were blocked by N-acetylation and C-amidation, respectively. This synthetic peptide has been proven to be safe and successful as a treatment for chronic otitis media in a clinical phase I/II trial [138].

As described by a recent study of Lima Fuscaldi et al. [139], the antimicrobial peptide LyeTx I from the venom of the spider *Lycosa erythrognatha* was shortened to three novel derivatives from LyeTx I (LyeTx I mn; LyeTx I mnΔK; LyeTx I mnΔKAc), and their toxicity and biological activity as potential antimicrobial agents were evaluated in vitro and in vivo. One shortened derivative LyeTx I mnΔK presented the best score between antimicrobial (↓ MIC) and hemolytic (↑ EC50) activities. In vivo data, obtained in a mouse model of septic arthritis induced by *Staphylococcus aureus*, showed that LyeTx I mnΔK was able to reduce infection and therefore may be the best candidate as an antimicrobial agent, due to its shorter amino acid sequence, lower toxicity, and its higher biological activity.

EeCentrocin 1 is a novel antimicrobial peptide obtained from the marine organism *Echinus esculentus*. The original molecule shows a heterodimeric structure with a large monomer containing an active heavy chain. This heavy chain has been used as a model to explore the structure–function relationship to optimize its antimicrobial activity. This goal has been achieved by truncating the heavy chain and replacing several nonessential amino acid residues. The resulting antimicrobial peptide showed more potent antifungal and less hemolytic activity with no relevant cytotoxicity. This constitutes a successful example to decrease the molecular size of an originally large antimicrobial peptide and in parallel ameliorate its pharmacological properties [140].

### 3.2. Introduction of Protective Groups, Non-Natural Amino Acids, and Cyclization: Combination of Derivatizations, and Hybrid Peptides

The protection of amino acid reactive positions, including the α-amino N, the amine groups, carboxylic acids, alcohols, and thiols, or the carboxylic terminus is a crucial challenge in peptide synthesis. It is essential to avoid polymerization to diminish unwanted reactions during the process. Adequate management of protecting groups during synthesis can improve the production efficiency and permits the preparation of complicated structures based on peptides. Thus, the spatial geometry of each protecting group is critical to obtaining adequate control of the molecular structure. It is very convenient to use unmasking procedures for exposing amine, carboxylic acid, alcohol, and thiol groups to properly synthesize peptides and associated molecules [141].

For example, a series of chemical modifications are available to protect a peptide sequence against proteolytic digestion. These include shielding the free N-terminus of a peptide, e.g., by acetylation, and the C-terminus by amidation. The naturally occurring alpha and L-amino acid residues can be displaced by their corresponding beta forms and their enantiomeric D-forms.

In a recent study of Wakabayashi et al. [142], N-acylated or D-amino acid peptide derivatives based on the sequence RRWQWRMKK in lactoferricin B demonstrated higher antimicrobial activities than those of lactoferricin B against bacteria and fungi. The most potent peptide, conjugated with an 11-carbon-chain acyl group, showed 2–8 times lower MIC than lactoferricin B.

Highly potent antimicrobial peptide derivatives of bovine cateslytin were described in a study by T. M. Postma and R. M. J. Liskamp [143]. Here, the authors described that the bovine cateslytin (RSMRLSFRARGYGFR) was significantly more potent than the human analog (SSMKLSFRARGYGF), as it contains two additional positively charged arginine residues. The C-terminal carboxylic acid was changed into a C-terminal amide. The methionine residue, which is often not critical for a peptide’s activity, was exchanged by the unnatural amino acid norleucine. The amphiphilic balance between charged and hydrophobic residues was fine-tuned by changing phenylalanine residues for tryptophan residues. Lastly, the amino acid serine was replaced by threonine to alter the amphiphilic balance by adding extra methyl groups. All these rational substitutions resulted in a ten-fold enhancement of antimicrobial activity, e.g., against *E. coli* and *S. aureus*, in a low micromolar range without an increase in potential hemolytic activity.

A very successful strategy to enhance peptide stability is the cyclization of a linear peptide sequence. This avoids the exposure of N- or C-terminal residues to exopeptidases. The most commonly used methods for peptide cyclization are head-to-tail cyclization and the introduction of disulfide bonds to stabilize the peptide backbone. Successful AMP therapeutics have been developed based on cycled structures, e.g., Gramicidin S [144], Daptomycin [145], and Theta-defensin [146].

On the other side, linearization of a cyclic active peptide such as bactenecin leads to the successful generation of new AMP drug candidates. Bactenecin, a 12-amino acid AMP found in bovine neutrophils [90], is the smallest known cationic AMP containing two cysteine residues forming a stable disulfide bond. This highly stable peptide potently binds to LPS and destroys the structure, fluidity, and permeability of the inner membrane, which finally results in the release of cytosol and subsequent death of the bacteria. Unfortunately, bactenecin showed significant hemolytic activity and cytotoxicity; therefore, new linear peptides were designed and showed enhanced antimicrobial activity but significantly lower cytotoxicity than those of bactenecin [147]. Based on the bactenecin sequence, promising innate defense regulators (IDRs) have been synthesized, combining the immunomodulatory and antimicrobial activities of classical AMPs [148].

Cyclic analogs have been prepared from linear hexapeptides derived from the sequence AcRRWWRF containing domains rich in arginine and tryptophan. Their conformation and their antimicrobial activity have been studied in detail [149]. A pronounced activity-improving and bacterial selectivity-enhancing effect was found upon their cyclization. The threshold values of low and high hydrophobicity have been determined during the process of cyclization. In this way, the cyclization of small peptide molecules with the proper amino acid composition was successfully used to design more effective antimicrobial peptides [149].

The expression of antimicrobial peptides in plants is a very promising strategy in order to circumvent the high costs of bulk synthetic procedures. However, their heterologous expression in a cost–benefit way demands a combination of derivatizations with some structural requirements such as reduced size, retention signals, and target sequences to help peptide detection. The modification of the antimicrobial undecapeptide BP100 presented ideal conditions for being expressed in plants after proper derivatization showing optimal biological properties [150]. Forty analogs were obtained by the introduction of repeated monomers into the original undecapeptide. Antimicrobial, hemolytic, and phytotoxic activities and protease susceptibility were tested with adequate parameters of acceptance. This derivatization strategy is very promising for the designing of plant-expressed antimicrobial peptides.

Another interesting aspect of optimization is the combination of active structural regions of two known antibiotic peptides. In the study of Klubthawee et al. [151], key structural and physicochemical parameters have been used in combination with rational engineering to design novel short α-helical hybrid peptides inspired by the well-known natural peptides, cathelicidin, and aurein. One combined peptide, PA-13, showed a remarkable broad-spectrum antibacterial activity, especially against *Pseudomonas aeruginosa* with no toxicity and maintained antimicrobial activity in the presence of physiological salts, and displayed rapid binding and penetration activity, which resulted in membrane depolarization and permeabilization. In addition, PA-13 showed an anti-inflammatory response via lipopolysaccharide (LPS) neutralization with dose-dependent, inhibiting, LPS-mediated Toll-like receptor activation. This study revealed the therapeutic potential of hybrid peptides in the rational design of novel AMPs.

The optimization of AMP sequences requires knowledge of their molecular mode of action. Recent advances in microscopy technology and cell biological labeling techniques allow studying mechanisms of AMPs in unprecedented detail. The review of Schäfer and Wenzel [152] gives an overview of available in vivo methods to investigate the antibacterial mechanisms of AMPs. They discuss global profiling techniques, such as genomic and proteomic approaches, as well as bacterial cytological profiling and other cell biological assays and cover recent approaches to determine the effects of AMPs on cell morphology, outer membrane, cell wall, and inner membrane properties, cellular macromolecules, and protein targets. They expand on methods to examine cytoplasmic membrane parameters, such as composition, thickness, organization, fluidity, potential, and the functionality of membrane-associated processes. These newly available methodologies enable one to study the mechanisms of AMPs in living bacteria, which allows one to design and study more potent and effective AMPs.

### 3.3. Structure–Activity Relationship (SAR) Analysis and In Silico Optimization of AMPs

The relation between peptide structure and antimicrobial activity is essential for improving both in vitro and in vivo therapeutic actions against pathogens and also for designing in silico new molecules. In this sense, phylloseptins, a family of AMPs identified in the skin secretions of tree frogs, showed interesting conserved structural features. Modified analogs were obtained by solid-phase synthesis, followed by RP-HPLC [153]. The modifications added in these analogs resulted in relevant changes of their physicochemical parameters such as hydrophobicity, hydrophobic moment, and net charge within the secondary structure. In addition, their antimicrobial action, membrane permeation, and cytotoxic effects have been successfully optimized compared to antibiotics currently used for the anti-infective therapy against methicillin-resistant *S. aureus* (MRSA).

Waghu and Idicula-Thomas [154] have summarized the growing in silico possibilities that provide the open-access resources for the optimizing and development of new potent AMPs on the basis of algorithms. For example, the collection of antimicrobial peptides (CAMP), CAMPSign, and ClassAMP have been developed to enhance research on antimicrobial peptides (AMPs), and machine learning-based predictive models are made available for users through these resources. The CAMPR3 has >10,000 sequence entries, 757 structures, and 114 family-specific signatures of AMPs. This database also provides tools for AMP sequence and structure analysis. CAMPSign employs family-specific sequence conservation in the form of patterns and hidden Markov models to identify AMPs. The ClassAMP data tool can be used to subdivide AMPs into antibacterials, antifungals, or antivirals according to their amino acid sequence. These online resources will advance the current understanding and development of AMPs.

## 4. Preclinical Development: Stability, Toxicity, and Half-Time

There is sufficient evidence to prove that long-term utilization of antibiotics develops drug resistance in pathogens. Many drug-resistant strains have been pointed as a result of sustained use (and abuse) of these conventional antibiotics [155]. Antimicrobial peptides (AMPs) exert a wide antimicrobial action and reasonably high antimicrobial activities [156]. The occurrence of these relatively small proteins offers a magnificent platform to develop promising antimicrobial peptide molecules in contraposition (or complement) of current chemical (conventional) antibiotics [157]. Several AMPs have been approved by the US Food and Drug Administration (FDA), but many of these peptides are frequently very questioned for clinical applications since they are not stable enough with a non-long half-life and a significant level of toxicity and hemolysis [158].

The multifunctional condition of natural AMPs induces one to think that they have not evolutionally developed as specialized anti-infective molecules [159]. A very important limitation is their contextual action [160]. Natural AMPs work only under a specific scenario in which there is an evolution of tissue interactions over millions of years [159]. In this sense, many natural AMPs are negatively influenced by serum, salt environment, and concentrations of divalent cations [160].

**Figure 3:**
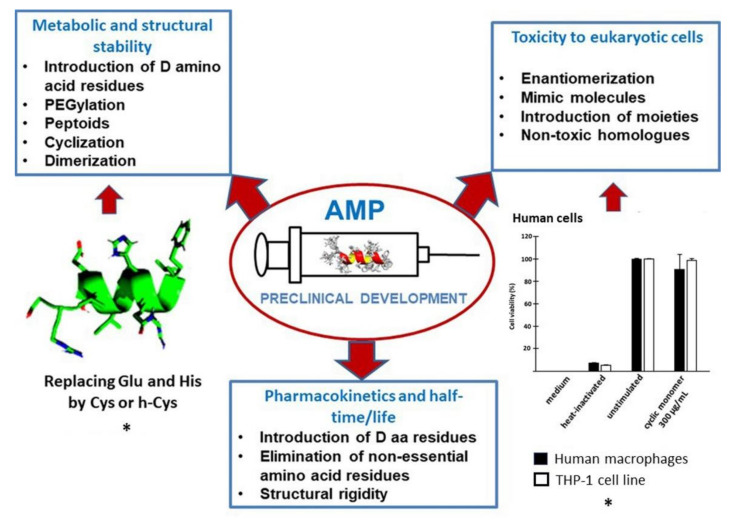
Some preclinical strategies for using antimicrobial peptides as therapeutic drugs. The figures marked with an asterisk were adapted from the publication *ACS Omega* [164]. Further permissions related to the material excerpted should be directed to the ACS.

A stable set of pharmacokinetic and pharmacodynamic parameters for AMPs is needed in terms of an adequate quality platform for their pharmaceutical utilization [161,162,163]. In this section, we focus on three of them: metabolic and structural stability, toxicity to eukaryotic cells, and pharmacokinetics and half-time/life (Figure 3).

### 4.1. Metabolic and Structural Stability

Some approaches to change the peptide molecular structure have been introduced to avoid the precarious metabolic stability of AMPs in vivo and decrease their susceptibility to protease cleavage. The incorporation of non-L-amino acids, end-conjugation by hydrophobic amino acid extensions, cyclic structures inside the molecule, and treatment of the N- and C-terminal extremes by N-acetylation, C-amidation, or N-pyroglutamate have been explored [165]. Beyond that, PEGylation is a powerful tool, where a polyethylene glycol chain is added to a biomolecule to optimize its pharmacological properties. It is one of the first and most extensively studied approaches for optimizing AMPs, e.g., magainin and the food preservative nisin [10].

There is a high number of possible modifications to design novel AMPs. Peptoids, having a displacement of the side chains to the nitrogen atom instead of the alpha carbons, produce a “modified” peptide bond, which is generally protected from proteolytic attacks, allowing the peptide a successful arrival to the site of infection [166].

A synthetic magainin peptoid has been reported to change the conformation of aromatic groups and to control the overall charge on the molecule. This new molecule was able to show activity against a lot of strains of *Staphylococcus aureus* and *Escherichia coli*, in comparison with the original magainin. Increased immunomodulation was also demonstrated for this magainin peptoid, including neutrophil chemoattraction and significant macrophage activation [167].

Antimicrobial peptides are often sensitive to serum proteolytic attack in high therapeutic doses. For that reason, they have been frequently accepted by regulator offices as topical antimicrobial agents [156]. For example, bacitracin and gramicidin are employed only for topical uses due to their protease degradation and strong hemolytic side effects. Nevertheless, local applications also represent particular challenges. Topical gels and creams require an effective penetration of the biomolecules into the tissue for being beneficial in skin lesions [168]. Therefore, drug delivery is a critical factor in the efficacy of antimicrobials. The therapeutic development of antimicrobial peptides requires an optimization of each sequence to choose the optimal delivery method. In this context, the synthetic modification of an antimicrobial peptide can increase its stability and allow for better drug-delivery options [169].

Cm-p5 is a peptide derived from a marine mollusk *Cenchritis muricatus* (Gastropoda: Littorinidae), that showed antifungal activity against human pathogens. This peptide had no toxicity against animal cell lines in vitro with an elucidated α-helical structure under conditions of cell membrane scenario and a random structure in polar media [170]. In principle, Cm-p5 is not a per se candidate for therapeutic agent due to its protease susceptibility conferred by two Arg residues in the structure and a free N-terminal. A covalent modification could increase its metabolic stability through a helical stabilization of the molecule that can play an essential role in its biological activity. The essential role of Glu and His residues in the helix stabilization was investigated, and two Cys substituted both amino acid residues in the original sequence. The resulting cyclic peptide improved the antimicrobial activity and could possibly enhance its pharmacological properties [164].

The therapeutic AMP WLBU2 peptide displays activity against multidrug-resistant bacteria for both planktonic and biofilm growing modes. A substitution of d-Val for l-Val decreased proteolytic susceptibility with increased activity against bacteria, mainly in biofilm growing mode [171].

### 4.2. Toxicity to Eukaryotic Cells

One of the crucial aspects for determining the usefulness of a biomolecule for therapeutics is its toxicity to animal cells in vitro and, lastly, in vivo. Since some AMPs may show deleterious activity against tumor cells and a putative anticancer therapy [172], toxicity against animal cell lines may not be suspicious for toxic behavior to nontumorigenic cells of the patient. In addition, to avoid proteolysis, as mentioned above, enantiomerization is a good option for diminishing toxicity for systemic applications and therapeutic purposes if, of course, this approach does not affect the antimicrobial potency [171].

The clinical introduction of AMPs requires an extensive evaluation of in vitro and in vivo toxicity to eukaryotic cells. Some AMPs have experimentally been nephrotoxic enough at therapeutic doses to be discarded or reevaluated after modifications [173]. In general, the differential toxicity of the AMPs is basically due to differences in the membrane composition or disposition between the animal cells and the pathogens [174]. In addition to their convenient selectivity, it has been reported that AMPs have a relatively low possibility to generate toxicity, both locally and systemically [175]. The majority of articles have reported no drastic consequences about toxicity in experimental animals assayed with AMPs. One of the major disadvantages of chemical antibiotics is the delivery of pathogen-associated molecular patterns (PAMPs), which can provoke septic syndrome and death, as in the case reported for ciprofloxacin [176]. In this sense, some AMPs were reported to be successful in preventing sepsis induced by microbial endotoxins in vitro and in an animal model [177].

On the other hand, colistin, a commercial antibiotic, is used as a last resort due to its nephrotoxicity. Specially designed drug delivery systems are needed to decrease the overall toxicity of any AMP therapy, which justifies why most antimicrobial peptides have been introduced as local applications. Synthetic mimics of AMPs constitute a new type of peptide anti-infective therapy. They have been rationally designed to keep an antimicrobial activity and, at the same time, a flexible chemical structure to keep the desirable characteristics as enhanced antimicrobial power with a minimum of cytotoxicity and proteolytic susceptibility. Synthetic mimics are useful to include noncanonical amino acids in the synthesis and more complicated spatial motifs [178]. Synthesis on solid phase is utilized to create many AMPs with the option of introducing specially designed moieties [179]. Compared to, e.g., recombinant production techniques, the use of solid-phase peptide synthesis (SPPS) for the generation of anti-infective molecules is less cost-intensive and the better approach concerning biosafety [169].

Modifications on peptide synthesis to decrease systemic toxicity allow peptides to be used inside the body. In this sense, changing the interactive membrane region of a peptide homolog reduced the cytotoxicity of animal cells [180].

### 4.3. Pharmacokinetics and Half-Time/Life

The mammalian physiology has evolved to eliminate foreign (possibly dangerous) proteins/peptides as soon as possible, with an integrated approach for the possibility that they could be toxic or from a pathogenic origin.

Long-active peptides are the “golden goal” of peptide/protein therapy. In this sense, strategies to extend plasma half-time of such biomolecules have been urgently demanded. Small plasma half-times are mainly caused by rapid and intense renal clearance and proteolytic cleavage during systemic application. Well-designed changes can be conducted to obtain an extension of plasma half-time. Having fewer amino acid residues and L-analogue amino acids replaced for D-amino acids, the plasma half-life of the peptide octreotide increased 1.5 h compared to somatostatin. A PEG2, 40 K conjugate of INF-α-2b increased 330-fold plasma half-time compared to native INF-α-2b [181].

The hormone insulin and analogs have a short half-life (from 4 to 6 min) in blood. Being the first peptide hormone produced by genetic engineering, this biomolecule was admitted by the FDA in the early 1980s to treat diabetes [182,183]. AMPs approved by FDA exhibited a longer half-life than insulin [7,162,184]. In this sense, daptomycin, oritavancin, dalbavancin, telavancin, and colistin have shown half-lives of 8–9 h, 14 days, 8 h, 195.4 h, and 5 h, respectively. The mean half-life of new drugs approved by the FDA is about 50 h (9 h, median), and the approval for small peptides for treatment is 37 h (3 h, median). The majority of peptides in this approved group are relatively stable in the body [161,163,185]. Peptides showing more structural rigidity may exhibit long half-lives [186,187,188]. Daptomycin and colistin (lipopeptides) and vancomycin, oritavancin, dalbavancin, and telavancin (cyclic lipoglycopeptides) show more stability than noncyclic versions [189]. The introduction of nonstandard amino acids into AMPs can avoid proteolytic cleavage and can prolong their half-time [190].

A generally applicable approach to improve the half-time of AMPs is to replace arginine residues with -amino-3-guanidino-propionic acid (Agp). In this sense, an arginine-rich short antimicrobial peptide, Sub3, was eliminated from mouse serum after 8 h, but when such substitution was made, degradation was diminished by 80% without any alteration of its antimicrobial activity [191].

Aiming to develop a practical and efficient utilization of peptides as therapeutic drugs mainly for systemic applications, the aspect of sustained half-life times has been favored with a manually curated resource of experimentally determined half-life of peptides. With more than 1000 entries, it is a valuable tool for knowing this crucial therapeutic parameter [192].

## 5. Peptide Release Systems (Conjugation, Liposomes, and Nanoparticles)

The absence of new antibiotics and the increased incidence and persistence of multiresistant germs require new antimicrobial strategies. In addition, the advent of emerging and re-emerging bacterial and fungal pathogens and new or mutated viruses such as Ebola, Zika, and, nowadays, SARS-CoV-2 pandemics constitutes a huge challenge to develop new and effective antimicrobial drugs [193].

Antimicrobial peptides (AMPs) are part of the defense strategy of virtually all naturally occurring organisms. They belong to the innate defense against infectious diseases, so-called “natural antibiotics”. They allow fast and efficient humoral innate immune responses, which are mainly composed of such peptides [10].

AMPs are capable of interacting with, perturbing, and destroying microbial membranes but, occasionally, also interacting with animal cell membranes in specific conditions; therefore, they can be employed as delivering vectors for a wide list of bioactive molecules [194]. Nevertheless, in addition to their antimicrobial potential, there is a dramatic claim for increasing in vivo killing or, at least, controlling microbial growth with enough efficiency and security. In this sense, some modifications are being conducted for functionalizing AMPs by conjugation [195].

Since 2015, the U.S. Food Drug Administration has approved more than 200 new drugs (about 150 new of chemical composition and 58 of biological origin). From them, 15 were peptides or molecules containing peptides (7% of the total number of drugs). For this therapeutic scenario, a peptide is a molecule with two or more amino acids coupled by a peptide bond and chemically synthesizable [196]. The use of peptide as therapeutic drugs has been almost neglected by pharmaceutical distributors due several limitations for their implementation [197]

**Figure 4:**
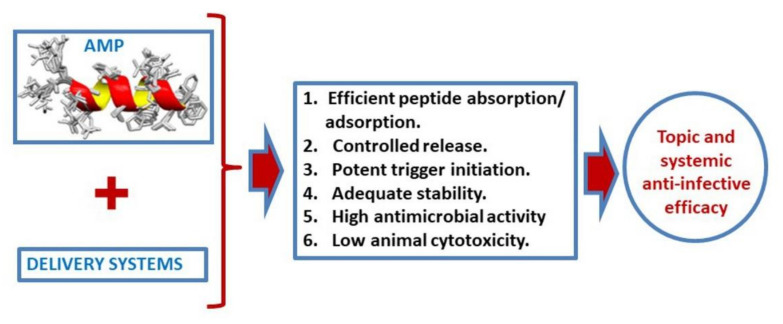
Some platforms and carriers for AMP release systems.

Efficient delivery systems for AMPs are prompted to play a significant role in developing strong and secure AMPs-based therapeutics (Figure 4). It must be achieved by reducing the possibility of chemical or biological cleavage from the formulation or after administration, by reducing adverse reactions, by managing AMP release rate, by promoting biofilm interaction, or through a colocalization with intracellular microorganisms [198]. The introduction of therapeutic peptides will be a reality in practical medicine in the near future [199].

**Figure 5:**
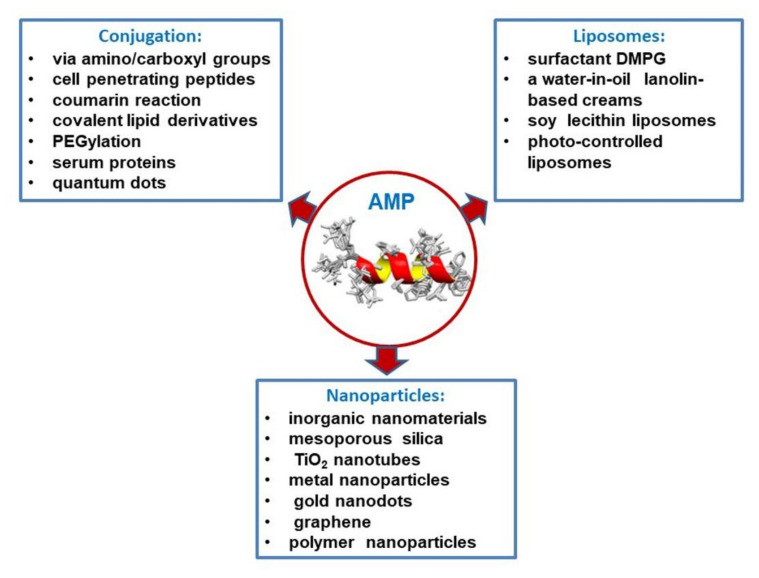
Desired general features for an efficient AMP delivery system.

Delivery systems play a crucial role in the transit of antimicrobial peptides from preclinical to clinical application [200]. The release carriers for AMPs can be grouped around the following items: synthetic polymers, complex carbohydrates, antibodies and proteins, DNA-based nanostructures, lipid capsules, metal nanoparticles, and other nanoparticles [201]. In this section, we focus on the following three topics (Figure 5): AMP conjugates, as well as nanoparticles and liposomes for AMP delivery.

### 5.1. Conjugation of Antimicrobial Peptides

Currently, antibiotics–peptide conjugates have been described as a product of a covalent junction of conventional antibiotics with an AMP [202]. In general, peptides can be functionally modified to obtain peptide conjugates by combining several molecules attached with some coupling procedures. A current trend is to fuse the molecular additive via the N-terminal alpha amino group, using a carboxyl group to be converted into an amide bond. This can be frequently achieved when the peptide is anchored to the solid matrix employing current reagents that are used in solid-phase peptide synthesis. Nevertheless, if the conjugate is intended to be obtained in a solution with the free peptide, a chemically selective coupling procedure should be employed via activation by amino reactive N-hydroxysuccinimides, thiol-reactive maleimide groups, copper (I)-catalyzed alkyne-azide cycloaddition, and some others [203].

The AMP magainin [88] was conjugated with the arginine-rich cationic cell-penetrating peptide R9 to enhance the antibacterial action. The resulting CPP-conjugated magainin provoked a four-fold increase in antimicrobial activity against Gram-positive species but demonstrated a 16-fold enhance against Gram-negative bacteria. Even when the CPP−AMP conjugate did not augment the membrane permeability, it clearly translocated across the lipid layer [204].

Using copper (1)-catalyzed alkyne-azide cyclo addition coumarin reaction, a benzopyrone exhibiting antimicrobial, anti-inflammatory, and anticancer activities was conjugated to Ubiquicidin. An antimicrobial peptide action was determined into a range of 0.04–0.18 mMol/L with very low toxicity [205,206].

The use of AMP conjugation for escaping peptide purposes and the subsequent endocytosis-mediated delivery have been documented for the case of the cell-penetrating peptide TAT11 with an AMP derived from cecropin A and melittin. A better endosomal release was observed in this sense [207]. On the other hand, the release of nucleic acids was demonstrated using a conjugate composed of the AMP C(LLKK)3C, fused to the TAT peptide and stearic acid in terms of its capacity to lyses endosomal membranes [208]. This approach could be used for a crosspresentation of the SARS-CoV-2 receptor binding domain antigens (RBD) to potentiate virus-specific CTL responses upon cytoplasmic presentation via MHC Class 1 to CD8 T lymphocytes.

A very attractive approach, but compromised in terms of inherent toxicity, is enhancing the activity of AMPs by improving their interaction with the bacterial surface/membrane with the addition of lipids [209]. This has been conducted with fatty-acid-charged AMPs in model membranes. In addition to subsequent toxicity due to the length of the fatty acid, an increased aggregation and self-assembly of the conjugates can be achieved [210]. This kind of lipid conjugates could be released inside a nonpolar environment for topical application on the site of infection.

Polyethylene glycol conjugation has been widely explored to increase the efficiency of therapeutic proteins systems. This so-called “PEGylation” has shown benefits as an increased time in blood circulation and a desirable diminishing in tissue uptaking by the reticuloendothelial environment. In the case of AMPs, PEGylation decreases proteolytic susceptibility and toxicity. Nevertheless, this advantage is frequently accompanied by losing antimicrobial activity [211]. Although PEGylation is the most popular conjugation approach for peptide therapeutics, other compounds, polymers, and proteins could be used for this purpose [201].

The chemical conjugation of the AMPs anoplin [212] and temporin L [213] to the glycopeptide vancomycin and its dendrimers was reported. This combination enhanced membrane disruption of large unilamellar vesicles only for the case of temporin L. On the other hand, the presentation via dendrimer conjugation enhanced vesicle disruption for anoplin and not in the case of temporin L [214].

To decrease microbial presence on polymer surfaces [215], AMPs can be directly implanted onto the polymer by a covalent bond. One β-sheet peptide and other α-helical peptide were immobilized on PEG-resin. Such conjugates were evaluated against bacterial species, but only the resin-containing β-sheet peptide resulted in antibacterial action [216].

Peptide dendrimers [217] of about 30 kDa (or more) were produced by ligation of multivalent chloroacetyl cysteine. Structural studies have shown molten globule states structures. Such dendrimers attracted to *P. aeruginosa* lectins and inhibited biofilm formation. In accordance with this multivalency, the amino acid composition of the dendrimers strongly influenced biological activity [218].

Nisin [35] was conjugated with hyaluronic acid and tested against *P. aeruginosa*, *S. aureus*, and *S. epidermidis*. The conjugate was found to display relevant action against these bacteria in solution, with a concentration-manner behavior [219].

Moreover, AMPs may be conjugated to serum proteins for improving their efficiency, as reported with the conjugation of the peptide BB28 [220] to an albumin domain (A) in its N-ending. The subsequent conjugate exhibited a stronger binding affinity for albumin than BB28, resulting in a larger circulation half-life in a couple of hours [221].

In the promising area of theranostics [222], some attempts at combining the detection and treatment of infections have been made utilizing quantum dots, even when some types of these preparations have shown toxicity in animal cells with obvious limitations as drug release system. Nevertheless, this platform has attracted the interest of AMPs researchers as, for example, the conjugation of BSA-stabilized ZnO quantum dot with the AMP fragment UBI29-41 and an indocyanine green-based near-infrared dye. These combinations decreased the cell toxicity and were able to differentiate microbial infections from nonmicrobial inflammation or tumors in vivo. These conjugates were ligated later to vancomycin or methicillin, with better antibacterial activity against MRSA strains compared to free methicillin, presumably due to an augmented permeation in the membrane [223]. The antimicrobial action and toxicity of quantum dots coated with or without indolicidin were explored with a related approach. Increased bactericidal activity was demonstrated with the peptide conjugation for *S. aureus*, *P. aeruginosa*, *E. coli*, and *K. pneumoniae*, and a low toxicity for the peptide-combined particles [224].

### 5.2. Liposome as Antimicrobial Peptide Delivery Tools

Liposomes and lipid bilayer constructs containing a nonpolar nucleus [225] have been utilized for years in drug delivery. The bilayer is mainly composed of compact phospholipids with some other lipids as cholesterol for stabilizing the structure. Because of their amphiphilic nature, the phospholipids spontaneously organize, in an aqueous solution, a bilayer with their polar head groups pointing outside/inside and their nonpolar fatty acid tails compacted at the interior [201]. Some advantages of such particles are biodegradability, lower toxicity, lower immunogenicity, a friendly scenario for surface modification, suitable for both hydrophobic and hydrophilic peptide passengers, protection from proteolytic cleavage, passive targeting to infection focus by the enhanced permeability, and retention effect. Some drawbacks: lower stability in vivo by phagocytic attacking, difficulties for sterilization in a large scaling set up, weak peptide capacity, restricted control for a specific delivery, and pseudoallergic adverse reactions during endovenous administration [226].

An important starting point for designing a liposome-based peptide material for delivery [227] is loading the cargo in (or on) the liposomal preparation. Poor peptide-loading efficiency disposes costly molecules and could diminish the therapeutic efficiency in both experimental and clinical approaches [228].

Earlier in 2000s, the encapsulation of polymyxin B [229] in surfactant-stabilized liposomes (DMPG) for aerosol delivery was proposed. Selected surfactant preparations were found to excerpts the best nebulizing application, avoiding decreasing liposomal integrity. Reasonable minimum inhibitory concentrations against *P. aeruginosa* could be demonstrated using a fast nebulizer starting in 2.5 mL [230].

In the area of cream and gel preparations for topical delivery to skin and mucosa, the LL-37-related peptide P60.4Ac [231] was included in three formulations: a water-in-oil lanolin-based cream (Softisan 649), an oil-in-water cream based on polyethylene glycol hexadecyl ether (Cetomacrogol), and hydroxypropyl methylcellulose (Hypromellose) 4000 gel. Antibacterial activity against mupirocin-resistant and -sensitive MRSA strains of S. aureus on epithelial and bronchial epithelial models were found to be smaller for the cream preparations than for the gel preparations. The latter killed almost 100% of the viable nonbiofilm bacteria and about 85% of the biofilm-related bacteria without any significant cytotoxic impact on keratinocytes and epithelial cells [232].

The influence of the molecular mass of lactoserum peptides [233] on their sequestering within liposomes made by soy lecithin has been investigated. While the peptide molecular mass was not relevant in the encapsulation efficiency, it clearly affected the encapsulation efficiency or liposome size with the same encapsulation efficiency [234].

Concerning peptide delivery and the possibility of proteolytic or chemical cleavage [235], it is crucial to consider the surface exposure with a relevant incidence on liposomes carrying peptides. In this sense, not only the encapsulation achievement or the liposome dimensions but also the specific peptide distribution on (or into) the liposomes need to be considered. In this regard, a photocontrolled release system using liposomes and the caged antimicrobial peptide temporin, activated by UV irradiation, has been successfully investigated [236].

### 5.3. Nanoparticle-Based Peptide Release Systems

Inorganic nanomaterials such as metal, metal oxide, silica, nanoclays, and platforms based in carbon have been investigated for macromolecular companying cargo [237]. They usually offer an appropriate defense from chemical and enzymatic cleavage, avoid aggregation and/or stereo-space modifications, permit a controlled material release rate, contribute to improved bioavailability, and keep a lower cytotoxicity. An additional advantage of such materials is their interaction with external magnetic fields and luminous radiation, which can be used for favoring the peptide delivery or provoking the generation of antimicrobial reactive oxygen species as a complement of the inherent peptide anti-infective activity. It is a good point for the nowadays popular incursion in the field of theranostic for simultaneously monitoring the detection of the pathogen, instead of aseptic inflammation, and at the same time, starting a therapeutic action [238,239].

Constituted by tunable pores in the nanomolar range, mesoporous silica is recommended for monitoring the delivery of porting molecules as peptides. This silica-based material has received attention because of its fine-defined pores, being able to permit the peptide loading and manageable delivery kinetics through controlling pore size, conformation, and working surface and chemistry. They have shown appropriate chemical stability, being relatively biocompatible, but depending on nanoparticle characteristic properties, dosage, and administration path [240,241,242]. In this sense, the influence of nanoparticle porosity and polarity of silica nanoparticles, including effects of charging and delivery of the mammalian antimicrobial peptide LL-37, have been studied regarding membrane interactions and antimicrobial activity [243].

The incorporation of LL-37 and chlorhexidine [231] into mesoporous silica monoliths has been achieved. The preparation was able to deliver both compounds significantly slowly enough to allow the incorporation of thiol groups into the wall pores. Moreover, the silica particles containing either the AMP or the low-mass antimicrobial compound showed strong bactericidal activity against *S. aureus* and *E. coli* with very low toxicity [244].

Silica-based nanoparticles [245] have also been used as a transporter of large antimicrobial proteins as lysozyme-coated mesoporous silica nanoparticles as antimicrobial agents. By enrichment of the surface, a high local enzyme concentration is allowed at the other charged nanoparticle area. A high local enzyme enrichment concentration is obtained in the proximity of the bacterial cell walls provoking peptidoglycan cleavage and membrane disorganization. The minimal inhibition concentration of the antimicrobial-loaded silica nanoparticles was found to act at a five-fold lower concentration than of free lysozyme in vitro. Using a mouse model of intestinal infection in vivo, the viability of bacteria in the colon after treatment resulted in three orders of magnitude lower than in the nontreated group [246].

The addition of a broad-spectrum short AMP into TiO_2_ nanotubes [247] increased antimicrobial activity on implant-associated infections. The procedure reduced the bacterial adhesion, and the capacity of TiO_2_ for crystallizing was found to influence the peptide delivery and the octahedrite behavior of TiO_2_. After the initial peptide delivery, octahedrite and amorphous TiO_2_ displayed similar desirable slow release times [248].

In the area of metal nanoparticles [249], aurum, argentum, platinum, and cuprum are the most used options for cargo delivery in both low-mass weight drugs and biomacromolecules, which can be properly adsorbed at these metal nanoparticles. Drug release can be produced by pH or ionic strength changes, elimination of the drug-surface thiol covalent bonds, or using light exposure with sensitive linking groups [250].

Gold nanodots (AuNDs) [251], with a procedure by etching and co-deposition of hybridized ligands, were useful to join surfactin and 1-dodecanethiol on gold nanoparticles. In comparison to surfactin itself, the surfactin-conjugated AuNDs were able to evidence an enhanced antimicrobial action against multidrug-resistant bacteria with lower cytotoxicity and hemolysis than surfactin alone. MRSA infection sites showed a more rapid healing, proper epithelialization, and more effective collagen fiber production in vivo [252].

Nanomaterials based on carbon derivatives [253], such as graphene and carbon nanotubes, are increasing the interest as drug release systems, even though they tend to aggregate in polar media and need stabilization by some surface changes [237].

A polyfunctional hybrid membrane, in which the antimicrobial peptide PGLa has been incorporated [254], and a carbon-nanotube-bridged graphene oxide conjugated to glutathione, was able to remove *E. coli*, and at the same time, toxic cations such as arsenic (III and V) and plumbous (II) [255].

On the other hand, indolicidin [256] has been conjugated to gold nanoparticles and carbon nanotubes. Both conjugates evoked complementary innate immune gene activation, and the indolicidin conjugated was able to protect against infection, reducing the bacterial charge [257].

In the area of polymer nanoparticle [258] carriers of AMPs, poly (lactide-co-glycolide) nanoparticles loaded with colistin were coated with chitosan or poly (vinyl alcohol) for the treatment of artificial cystic fibrosis mucus. Chitosan-coated and colestin-loaded particles were active against a biofilm of *P. aeruginosa* in comparison with colistin, alone [259].

In general, in the field of peptide release systems, some aspects remain without an exhaustive comprehension, dealing with peptide loading in or out from some release platforms. An issue that needs to be addressed is the reduction of steps mainly for physicochemical loading and releasing instead of covalent binding, and a prompting matter of attention is the scaling up of pilot and industrial plants. In vivo designs need to be increased in terms of bloodstream circulation time, tissue distribution, clearance, and other pharmacokinetic aspects [198,260])

As a manner of conclusion, some delivery systems have been explored as AMP release delivery tools. Many of them are well-established drug delivery systems, such as biomaterials and other materials less frequently employed in pharmaceutical formulations. Managing control peptide exposure and release and protecting peptides from proteolytic cleavage as cargo are urgent, as well as the control of unwanted peptide side-effects in vivo.

A more aggressive approach is needed if the peptide therapists aspire to an extensive introduction of AMPs into systemic treatments much more than the initial and still timid incursion as topic therapy in the anti-infective treatments.

## 6. Clinical Applications of Antimicrobial Peptides

The manufacture of peptides is relatively expensive; therefore, AMP production at a large scale has focused on obtaining AMP with a short sequence to minimize costs. In addition, peptides are extensively hydrolyzed in the gastrointestinal tract; therefore, it is necessary to consider suitable formulations and routes of administration for minimal degradation. Some peptides seem very promising in the preclinical stage but fail the clinical trials [261,262,263,264]. Thus, it is not surprising that part of AMP research has been guided to obtain topical antimicrobial peptides in a safer and cost-effective alternative.

Despite the limitations of antimicrobial peptides to reach clinical trials, some of them became very promising and paved their way to the market. In this section, we discuss some examples and their clinical applications (Table 1).

### 6.1. AMP with Antibacterial Effects

Bacitracin is one of the first known topical antibiotics; it was isolated in 1943 from *B. subtilis* var. licheniformis and then approved by the FDA in 1948 against Gram-positive bacteria (Table 2). Its mechanism of action involves the inhibition of cell wall and peptidoglycan synthesis; however, the development of resistance to bacitracin is uncommon. bacitracin’s parenteral administration can cause severe nephrotoxicity; therefore, it is used topically in combination with neomycin and polymyxin B to treat minor skin injuries [267,268,269].

Another example is the family of antimicrobial glycopeptides that acts by inhibiting cell wall synthesis through binding strongly to D-Ala-D-Ala and crosslinking the peptidoglycan chains. This characteristic makes them active only against Gram-positive bacteria as large molecules cannot pass through the LPS layer of Gram-negative bacteria, [270,271]. Vancomycin is one of the first-generation members of this family, a natural product from *Streptomyces* spp., which is commonly administered intravenously, although intraperitoneal, intraventricular, intrathecal, and intravitreal routes can also be used. Oral administration is less common due to its much lower bioavailability, which is below 10% [271,272].

Teicoplanin is another first-generation glycopeptide antibiotic, which requires less frequent dosing than vancomycin and can be administered intramuscularly. Teicoplanin is used to treat septicemia, endocarditis, and cystitis caused by multidrug-resistant enterococci. Bacteria that are resistant to vancomycin are also resistant to teicoplanin [270,271,273].

Regarding second-generation glycopeptide antibiotics, three new drugs have been approved: Telavancin, Oritavancin, and Dalbavancin. Telavancin is used for the treatment of pneumonia acquired in hospitals, and complicated skin infections; it has an extra mechanism of action by acting on cell membrane, causing depolarization of the cell [274]. Oritavancin is a semisynthetic lipoglycopeptide active against Gram-positive bacteria, MRSA, VRSA, and cocci resistant to vancomycin. Oritavancin can also kill bacteria by interrupting cell wall functions; it is used to treat complicated skin infections [275,276,277]. Dalbavancin is chemically derived from A-40926, a natural compound produced by the actinomycete Nonomuria spp [278]. Dalbavancin is active against a broad range of Gram-positive bacteria. Its prolonged half-life simplifies the dosing scheme through a once-weekly use and reduces healthcare costs, including hospital stay costs [279]. In addition, in contrast to other glycopeptides, the use of Dalvabancin has not been linked to nephrotoxicity. Its broad spectrum of activity against Gram-positive bacteria includes MSSA and MRSA; also, it has no drug–drug interactions [270,271,278].

Another family of peptide antibiotics is composed of lipopeptides. One of the most known members is Daptomycin, a cyclic lipopeptide extracted from *Streptomyces roseosporus* by fermentation. Daptomycin is used against aerobic and anaerobic Gram-positive bacteria, MRSA, and cocci resistant to vancomycin. It is approved by the FDA for many clinical uses such as cSSSI and *S. aureus* bacteremia, and it is also used in many off-label applications such as diabetic foot infections, *Staphylococci* joint infection, osteomyelitis and others. Its mechanism of action involves depolarization of the cell membrane, which leads to the disruption of intracellular functions such as DNA, RNA, and protein synthesis. A drawback of its use is the interactions with HMG-CoA reductase inhibitors; therefore, caution should be taken by monitoring the creatinine phosphokinase every week. In addition, Daptomycin cannot be used to treat pneumonia since it is inhibited by pulmonary surfactant [280,281,282,283].

Polymyxin B is among well-known natural peptides acting against Gram-negative bacteria; it neutralizes bacterial endotoxins by binding to the bacterial lipopolysaccharides (LPS) before the binding between LPS and monocytes. Polymyxin B is known for its nephrotoxicity and neurotoxicity since it acts as a cationic detergent by disrupting cell membrane leading to cell death. Therefore, it is generally recommended to use topically, although in some cases, histamine release symptoms are developed. Polymyxin is available in a triple combination of antibiotics and corticosteroids to treat eye and skin infections [284,285,286,287].

Colistin (also known as polymyxin E) has a broad spectrum of activity against Gram-negative bacteria. Like polymyxin B, colistin causes high toxicity to neurons and kidneys; therefore, the concomitant use of other nephrotoxic drugs should be avoided. Colistin is composed of two cyclic peptides, Colistin A and B, which interact with lipid A of the LPS because of the strong positive charge and the acyl chain. Colistin is used in multidrug-resistant lung infections and cystic fibrosis (CF); thus, inhalation and intravenous are the commonly used routes of administration, respectively. However, parental use is limited to those cases when no other option is available, due to the risk of toxicity [288,289,290,291,292].

Antimicrobial peptides Gramicidin D and S, from *Bacillus brevis*, can kill almost all Gram-positive bacteria and some Gram-negative bacteria plus some fungi. These AMPs are used topically to treat eye and nose infections. Gramicidin D and S are considered ionophores as they bind to the membrane of the bacteria to form pores, leading to extensive efflux of the ions and solutes and disrupting intracellular functions. Gramicidin D consists of gramicidin A, B, and C. Interestingly, gramicidin A also shows anticancer activity by inducing metabolic dysfunction and energy depletion, which leads to suppression of cell growth and angiogenesis in renal cell carcinoma [293,294].

### 6.2. AMP with Antiviral Effects

Hepatitis C is an infectious disease caused by the hepatitis C virus (HCV), which is an epidemic in some countries. Hepatitis C may lead to complications such as chronic hepatitis, hepatocellular carcinoma (HCC), and liver cirrhosis, which represent an economic burden on health care systems. Its impact of health and economy has lead efforts toward finding a cure to eradicate the virus infection and to avoid life-long treatments [295].

Telaprevir (TPV) is a peptidomimetic belonging to the first-generation HCV-protease inhibitors for the treatment of chronic hepatitis C; this oral drug inhibits the release of nonstructural viral proteins. Telaprevir was approved against chronic genotype 1 hepatitis C and is used in a triple combination with pegylated-interferon α and ribavirin. It has some side effects such as severe anemia (<10.0 g hemoglobin/dl) and rash. In addition, telaprevir inhibits cytochrome P450 3A and P-glycoprotein, resulting in interactions with coadministered drugs such as cyclosporine, tacrolimus, antihypertensive drugs, antidepressants, oral contraceptives, and antipsychotics. Considering its high cost, side effects, and limited activity only against genotype 1 HCV, its use is restricted to unresponsive patients [300,301,302].

Boceprevir (BOC) is also a direct-acting antiviral peptidomimetic and a first-generation protease inhibitor approved to be used the same way as telaprevir in a triple combination therapy with pegylated interferon and ribavirin [295,302].

Enfuvirtide is an antiviral peptide approved by FDA in 2003 for the treatment of HIV-1 infection. Enfuvirtide blocks the fusion of the virus with the host cells by binding to the gp41 heptad-repeat-1 (HR1) region, which is essential for the entry of HIV-1. Enfuvirtide represents the first HIV fusion inhibitor. Among its limitations is poor oral bioavailability due to rapid hydrolysis in the gastrointestinal tract; therefore, it is administered twice daily as a subcutaneous injection. Due to being very expensive, it is used only in patients unresponsive to other antiviral drugs or those with no good prognosis [303,304,305,306].

Atazanavir is an azapeptide and protease inhibitor, specifically for the treatment of HIV-1 infection. It is orally administered on a once-daily dosing scheme with didanosine and stavudine. This combination causes a rapid decrease in the viral count in treatment-naive patients after 48 weeks. Atazanavir is a substrate, inhibitor, and inducer of CYP3A4; therefore, it may cause some drug–drug interactions with other substrate, inhibitors, and inducers of CYP3A4. It is taken with food, and it needs low PH for its absorption; therefore, is contraindicated with proton-pump inhibitor and antacids [305,307,308].

In November 2010, Tesamorelin was approved as the first and only drug for the treatment of HIV-associated lipodystrophy. HIV-associated lipodystrophy is the deposition of fats in different body areas, mainly in abdominal region, as a secondary effect of antiretroviral therapy (ART). Tesamorelin stimulates the synthesis of growth hormone as it is a synthetic analogue of growth hormone releasing factor. It is administered by subcutaneous injection, resulting in reduction in the trunk fat, waist circumference, and improvement of other body image parameters. As a category X drug, it cannot be used during pregnancy [309,310,311].

### 6.3. AMP with Antifungal Effects

Antimicrobial peptides are not only promising as antivirals, antibacterials, and antiparasitic drugs but also as antifungal agents. In this section, we refer to three antifungal peptides in the market, which have shown excellent results in clinical application against candidiasis and other diseases caused by fungi [312].

The first example to discuss is caspofungin, the first discovered member of the family of echinocandin antifungals. Caspofungin inhibits the synthesis of the fungal cell wall by blocking beta (1,3) D-glucan synthase. It is mainly used to treat invasive aspergillosis and esophageal candidiasis. The main benefit of capsofungin over the classical antifungal agent amphotericin B lies in its lower side effect and lower drug–drug interactions as capsofungin is not an inducer nor inhibitor of CYP450. In addition, it shows good effects in the treatment against infection produced by *Aspergillus spp.*, *Candida spp.*, and *H. capsulatum*, in addition to those strains of *Candida albicans* that are resistant to azoles but are susceptible to caspofungin [312,313,314]. Given its antifungal properties and mechanism, capsofungin represents a suitable template for the development new antifungal candidates [315].

Micafungin is another member of this family; it is a lipopeptide with the same mechanism of action as capsofungin, low side effects, and a linear elimination kinetics [316,317].

Anidulafungin, a member of the echinocandin family, has the same benefits of this family which includes low side effects such as some rash, nausea, headache, and vomiting. Other advantages lie in its low drug–drug interactions as it is neither an inhibitor nor inducer of CYP 450, spontaneous degradation, same efficacy as fluconazole against candidiasis, high potency as broad spectrum antifungal, and its extended half-life of 18 h, which is the longest among antifungal member of this family [312,313,314,318].

## 7. Conclusions

The resurged interest in natural antimicrobial peptides as templates for the development of novel therapeutic agents has led to the implementation of strategies to make them suitable for systemic application in humans. In the present review, we summarized some of the current trends, such as chemical modifications and peptide delivery systems. In addition, some aspects of AMP preclinical development were discussed; and several examples of clinical applications were shown.

None of the current trends based on known AMP are exempt from challenges. In this sense, the continuous evolution of computational methods aided by an increasing understanding of AMP mechanisms of action, including the discovery of novel molecular targets, will significantly impact the design of novel AMP derivatives, conjugates and delivery systems.

On the other hand, nature still has much to offer in the search for novel AMP templates. The use of alternative AMP sources such as so-far “uncultivable” bacteria will undoubtedly provide more possibilities to the biological evaluation of AMPs and the extraction of unexplored molecules. In addition, proteomic/genomic technological advances combined with data mining, AMP prediction, and virtual screening will continue increasing the number of molecular structures to study and optimize on the way to new therapeutic AMPs. 

## Figures and Tables

**Table 1:** Antibacterial, antiviral, and antifungal peptides approved by the FDA since 1999 until 2019 [265,266].

Drug Name	Active Ingredients	Peptide	Applications
Orbactiv	Oritavancin diphosphate	Lipoglycopeptide	Treatment of Gram-positive bacteria causing complicated skin and skin structure infections (cSSSI).
Dalvance	Dalbavancin hydrochloride	Lipoglycopeptide	Treatment of Gram-positive bacteria causing complicated skin and skin structure infections (cSSSI).
Incivek	Telaprevir	Chemically modified peptide	Chronic hepatitis C treatment
Egrifta	Tesamorelin acetate	Synthetic peptide	Human immunodeficiency virus (HIV) treatment
Vibativ	Telavancin hydrochloride	Lipoglycopeptide	Treatment of Gram-positive bacteria causing complicated skin and skin structure infections (cSSSI).
Eraxis	Anidulafungin	Lipopeptide	Antifungal drug
Cubicin	Daptomycin	Cyclic lipopeptide	Treatment of Gram-positive bacteria causing complicated skin and skin structure infections (cSSSI).
Cubicin rf	Daptomycin	Cyclic lipopeptide	Treatment of Gram-positive bacteria causing complicated skin and skin structure infections (cSSSI).
Reyataz	Atazanavir sulfate	Azapeptide	Human immunodeficiency virus (HIV) treatment
Fuzeon	Enfuvirtide	Synthetic peptide	Human immunodeficiency virus (HIV) treatment
Cancidas	Caspofungin acetate	Cyclic lipopeptide	Antifungal drug

**Table 2:** Summary of antibacterial peptides in the market.

Drug	Type of Bacteria	Mechanism of Action	Use	Half-life	Route of Administration	Reference
Bacitracin	Gram-positive	Inhibits cell wall synthesis	Skin infections	1.9 days	Topical Ophthalmic Intramuscular	[267]
Dalbavancin	Gram-positive	Inhibits cell wall synthesis	Skin infections	6–10 days	Intravenous	[278]
Daptomycin	Gram-positive	Membrane lysis	Skin infections	9 h	Intravenous	[280,296]
Colistin	Gram-negative	Membrane lysis	Multidrug-resistant Gram-negative infections	3.0 ± 0.6 h	Intravenous	[297]
Gramicidin D	Gram-positive, some Gram-negative	Membrane poration/lysis	Skin and eye infection	Not Determined	Topical Ophthalmic	[293]
Oritavancin	Gram-positive	Membrane lysis and inhibits cell wall synthesis	Skin infections	Long serum half-life (393 h)	Intravenous	[276]
Polymyxin B	Gram-negative	Membrane lysis	Urinary tract and bloodstream infections	11.5 h	Ophthalmic Topical Intravenous	[286,287]
Teicoplanin	Gram-positive	Inhibits cell wall synthesis	Serious Gram-positive infections	Long half-life of approximately 47 h	Intramuscular Intravenous	[271]
Telavancin	Gram-positive	Membrane lysis and inhibits cell wall synthesis	Skin infections	8 h	Intravenous	[298,299]
Vancomycin	Gram-positive	Inhibits cell wall synthesis	Serious Gram-positive infections	7 to 12 h according to age	Oral Intravenous	[271,272]

## Data Availability

Not applicable.
